# Induction of hair follicle dermal papilla cell properties in human induced pluripotent stem cell-derived multipotent LNGFR(+)THY-1(+) mesenchymal cells

**DOI:** 10.1038/srep42777

**Published:** 2017-02-21

**Authors:** Ophelia Veraitch, Yo Mabuchi, Yumi Matsuzaki, Takashi Sasaki, Hironobu Okuno, Aki Tsukashima, Masayuki Amagai, Hideyuki Okano, Manabu Ohyama

**Affiliations:** 1 Department of Dermatology, Keio University School of Medicine 35 Shinanomachi, Shinjuku-ku, Tokyo, 160-8582, Japan; 2 Department of Physiology, Keio University School of Medicine 35 Shinanomachi, Shinjuku-ku, Tokyo, 160-8582, Japan; 3 Department of Biochemistry and Biophysics, Graduate School of Health Care Sciences, Tokyo Medical and Dental University, 1-5-45 Yushima, Bunkyo-ku, Tokyo, 113-8510, Japan; 4 Laboratory of Tumor Biology, Department of Life Sciences, Faculty of Medicine, Shimane University, Shiojicho 89-1, Izumo-shi, Shimane, 693-8501, Japan; 5 KOSÉ Endowed Program for Skin Care and Allergy Prevention, Keio University School of Medicine, 35 Shinanomachi, Shinjuku-ku, Tokyo, 160-8582, Japan; 6 Department of Dermatology, Kyorin University School of Medicine, 6-20-2 Shinkawa, Mitaka-shi, Tokyo, Japan

## Abstract

The dermal papilla (DP) is a specialised mesenchymal component of the hair follicle (HF) that plays key roles in HF morphogenesis and regeneration. Current technical difficulties in preparing trichogenic human DP cells could be overcome by the use of highly proliferative and plastic human induced pluripotent stem cells (hiPSCs). In this study, hiPSCs were differentiated into induced mesenchymal cells (iMCs) with a bone marrow stromal cell phenotype. A highly proliferative and plastic LNGFR(+)THY-1(+) subset of iMCs was subsequently programmed using retinoic acid and DP cell activating culture medium to acquire DP properties. The resultant cells (induced DP-substituting cells [iDPSCs]) exhibited up-regulated DP markers, interacted with human keratinocytes to up-regulate HF related genes, and when co-grafted with human keratinocytes *in vivo* gave rise to fibre structures with a hair cuticle-like coat resembling the hair shaft, as confirmed by scanning electron microscope analysis. Furthermore, iDPSCs responded to the clinically used hair growth reagent, minoxidil sulfate, to up-regulate DP genes, further supporting that they were capable of, at least in part, reproducing DP properties. Thus, LNGFR(+)THY-1(+) iMCs may provide material for HF bioengineering and drug screening for hair diseases.

Complex interactions between defined cellular subsets underline the processes of organogenesis and tissue regeneration^1^,^2^,^3^. In particular, ectodermal appendages, including hair follicles (HFs), mammary glands, and teeth, are formed via well-coordinated crosstalk between inductive mesenchymal and receptive epithelial cell populations^1^,^2^,^3^,^4^,^5^. Their ease of accessibility has made HFs attractive for investigation into morphogenesis and regeneration processes^5^,^6^,^7^. A great deal of evidence suggests that the dermal papilla (DP), a specialised mesenchymal component located at the proximal end of the HF, plays key roles in HF morphogenesis and regeneration^2^,^8^,^9^.

Experimental regeneration of HFs has attracted interest, as it enables a better understanding of skin biology, the development of models for drug discovery, and may eventually provide replacement therapy for intractable hair loss disorders, including scarring alopecia^9^,^10^,^11^,^12^,^13^. The biological characteristics of DP cells, including global gene expression profiles and biomarkers for hair-inductive capacity, have been well-studied in both mice and humans^7^,^14^,^15^,^16^. A large number of intact murine DP cells can be isolated for HF regeneration assays using cell surface markers represented by CD133^17^. However, in the case of human DP (hDP) cells, a methodology for efficient isolation and *in vitro* expansion capable of maintaining their intrinsic properties has not yet been fully established^7^,^16^. Therefore, preparation of alternate mesenchymal cell sources with trichogenic activity would be an attractive strategy for HF bioengineering.

Recently, a subset of human bone marrow-derived cells marked by high levels of LNGFR (CD271), THY-1 (CD90) and VCAM-1 (CD106) expression was found to exhibit properties of multipotent bone marrow stromal cells^18^,^19^ including rapid colony expansion, robust multilineage differentiation and self-renewal potency^19^. In addition, these cells show minimal expression of *P16INK4a in vitro*, indicating genetic stability and resistance to cellular senescence, clearly demonstrating the advantage of using this subset for the generation of specific dermal cell subpopulations, including DP cells. However, the LNGFR(+)THY-1(+)VCAM-1(hi+) subset accounts for less than 0.1% of collected living bone marrow cells, currently limiting their use for downstream applications^19^. Human induced pluripotent stem cells (hiPSCs) have a great deal of promise as material for regenerative medicine^20^,^21^. Induction of hiPSCs into mesenchymal cells with mesenchymal stem cell (MSC)-like plasticity has been demonstrated^22^,^23^, suggesting that induced mesenchymal cells (iMCs) analogous to LNGFR(+)THY-1(+)VCAM-1(hi+) bone marrow-derived cells may be generated from hiPSCs.

In the present study, we attempted to induce dermal cells with DP cell properties using iMCs phenotypically and functionally similar to a mesenchymal stromal cell subpopulation isolated from bone marrow^19^. The results of this study suggest that a recently conceived approach to regenerate DPs from patient-derived hiPSCs^24^ may be feasible. This study also supports the potential use of hiPSCs for the generation of tissue-inductive mesenchyme with the capacity for crosstalk with cells of epithelial lineage.

## Results

### Induction of iMCs from hiPSCs

**Figure 1:**
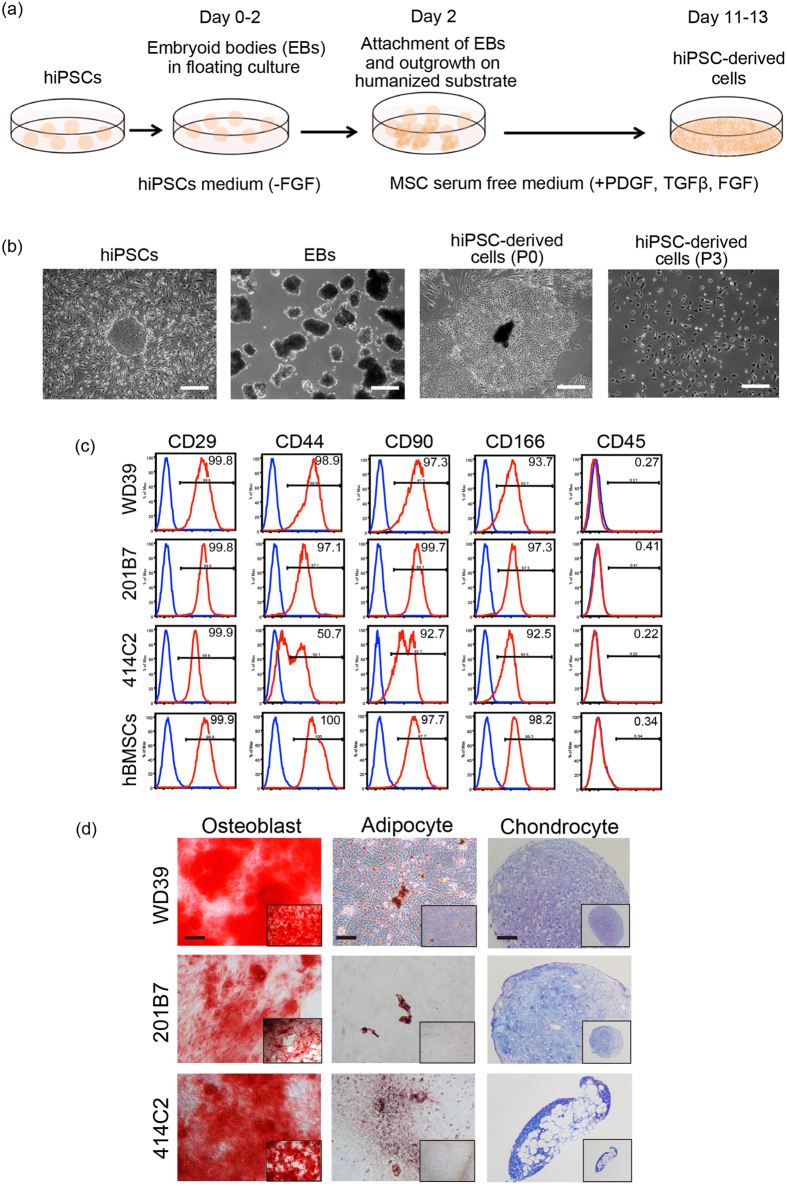
Generation of iMCs. **(a)** Schematic illustrating the established protocol. **(b)** Morphological characteristics of hiPSC-derived cells (WD39) during the course of differentiation and serial passage. Scale bar = 100 μm. **(c)** The results of flow cytometric analysis of hiPSC-derived cells after the mesenchymal differentiation protocol (red line). Isotype controls are used as negative controls (blue line). **(d)** The cells programmed from all tested hiPSC lines showed the capacity to differentiate into osteoblast, adipocyte and chondrocyte lineages indicating successful generation of induced mesenchymal cells (iMCs) with *in vitro* plasticity similar to that of hBMSCs. Note that WD39-derived cells were more efficiently differentiated into the three lineages. Scale bar = 100 μm. hiPSCs, human induced pluripotent stem cells; EB, embryoid body; FGF, basic fibroblast growth factor; MSCs, mesenchymal stem cells; hBMSCs, human bone marrow stromal cells; PDGF, platelet-derived growth factor; TGF-β, transforming growth factor-beta.

The hiPSC lines generated using the retroviral vectors 201B7^25^ and WD39^26^ and the integration-free episomal vector 414C2^27^ were examined for their capacity to differentiate into cell populations demonstrating mesenchymal properties. We established a novel protocol involving embryoid body (EB) formation, two days of floating culture, subsequent seeding onto a humanised substrate, and culture in MSC serum-free medium (MSC-SFM) containing PDGF, TGF-β, and FGF, reported to promote MSC proliferation and maintenance^28^. EBs attached rapidly and outgrowths of spindle-shaped cells reached confluence after 9–11 days of culture in MSC-SFM. Resultant hiPSC-derived cells could be maintained by passage (>passage 4) onto humanised substrate in MSC-SFM or onto plastic culture vessels in human MSC medium (Fig. 1a,b and Supplementary Materials and Methods).

Flow cytometric analyses of hiPSC-derived cells and human bone marrow stromal cells (hBMSCs) demonstrated near-uniform expression of fibroblastic mesenchymal cell markers^19^,^29^ integrin β1 (CD29), CD44, CD90 and CD166, with the exception of moderate CD44 expression in 414C2-derived cells (Fig. 1c, Table 1). HLA-DR, CD45, and CD31 were not expressed in hiPSC-derived cells (Fig. 1c and data not shown). Subsequently, hiPSC-derived cells were cultured under established conditions, allowing BMSCs to differentiate into osteoblasts, adipocytes and chondrocytes. The cells derived from all tested hiPSC lines exhibited the capacity to differentiate into these lineages, as indicated by positive staining for markers of the respective lineages (Table 1). WD39-derived cells were induced to differentiate into three lineages more efficiently than 201B7- or 414C2-derived cells (Fig. 1d, Table 1). These findings indicate successful programming of hiPSCs into iMCs with *in vitro* plasticity similar to that of hBMSCs^18^.

### LNGFR(+)THY-1(+) subset represents proliferative and multipotent iMCs

**Figure 2:**
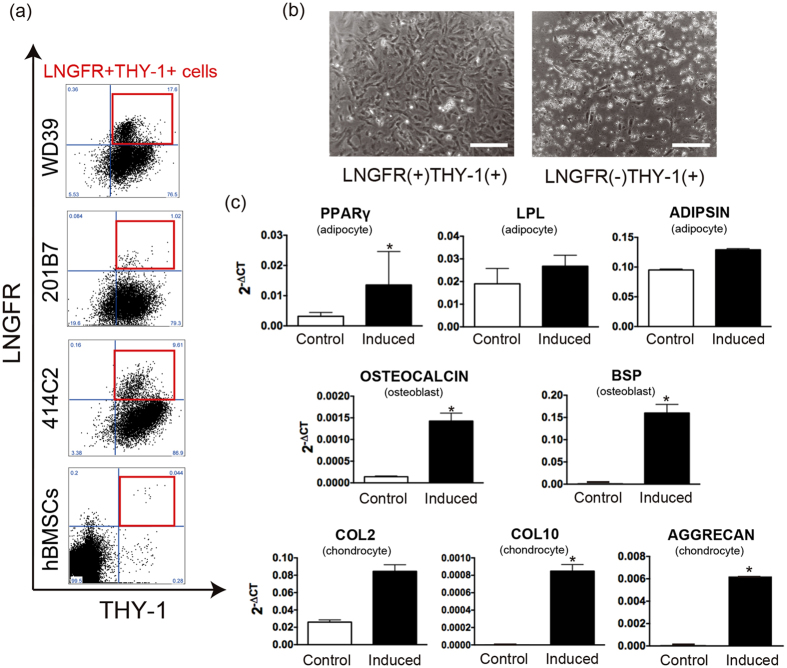
LNGFR(+)THY-1(+) cell population enriched in iMCs represents a proliferative and multipotent subset. (**a**) Flow cytometric analysis of iMCs. hiPSC-derived cells included an LNGFR+THY1+subpopulation. The incidence of LNGFR(+)THY-1(+) cells was much higher in iMCs than in hBMSCs. Note that all three hiPSC lines gave rise to LNGFR(+)THY-1(+) subsets. **(b)** Sorted LNGFR(+)THY-1(+) cells could be serially passaged, while LNGFR(−)THY-1(+) cells did not survive passaging. Scale bar = 20 μm. (**c**) LNGFR(+)THY-1(+) iMCs were able to differentiate into three mesenchymal lineages, as indicated by the up-regulation of respective lineage markers. *P < 0.05 for (**c**). Data presented in (**b**) and (**c**) were obtained with the WD39 hiPSC-line. hBMSCs, human bone marrow stromal cells.

The LNGFR(+)THY-1(+)VCAM-1(hi+) subset represents a small (<0.1%) but highly multipotent fraction of hBMSCs^19^. As most LNGFR(+)THY-1(+) cells expressed VCAM-1^19^, we focused on LNGFR and THY-1 expression profiles. Intriguingly, iMCs contained greater numbers of LNGFR(+)THY-1(+) cells (6.4% ± 2.97%–14.52% ± 2.06%) than did cultured hBMSCs (Fig. 2a, Table 1). The purities of isolated LNGFR(+)THY-1(+) and LNGFR(−)THY-1(+) iMCs were 82% ± 1.8 and 97% ± 0.6%, respectively, indicating successful isolation. Sorted LNGFR(+)THY-1(+) iMCs were serially passaged on plastic culture vessels in hMSC medium over four generations, while LNGFR(−)THY-1(+) iMCs were unable to be passaged after initial seeding (Fig. 2b). This difference suggests a higher proliferative capacity of LNGFR(+)THY-1(+) iMCs. Under osteoblast, adipocyte and chondrocyte differentiation culture conditions, LNGFR(+)THY-1(+) iMCs showed up-regulation of genes of the respective lineages (Fig. 2c), although their differentiation potential was inferior to their bone marrow counterparts based on the increase in lineage-specific gene expression following induction (Supplementary Fig. 1). In addition, LNGFR(−)THY-1(+) iMCs died out during induction. Therefore, we used LNGFR(+)THY-1(+) iMCs for the generation of dermal cells with DP cell properties.

### Programming of LNGFR(+)THY-1(+) iMCs into cells with DP properties

**Figure 3:**
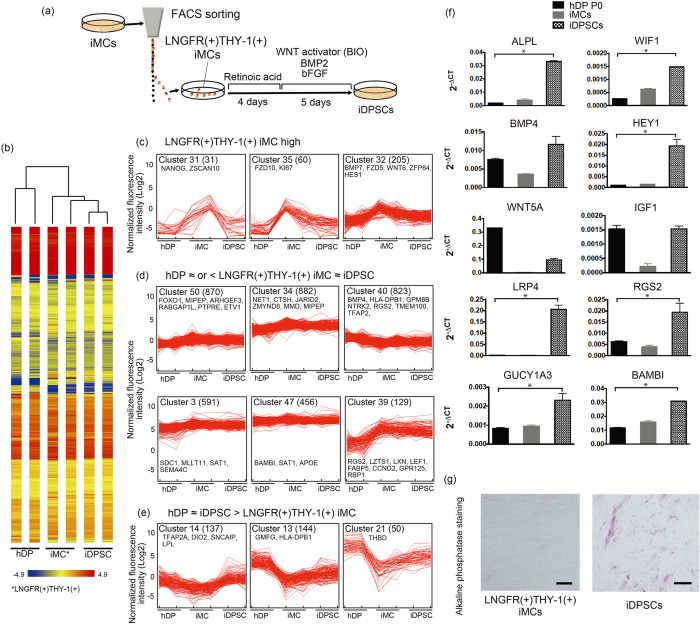
Induction of DP fate in LNGFR(+)THY-1(+) iMCs. (**a**) Summary of the DP induction protocol. Sorted LNGFR(+)THY-1(+) cells were exposed to retinoic acid (RA). Subsequently, the cells were cultured under dermal papilla activation culture (DPAC) conditions supplemented with WNT, BMP, and FGF signalling activators to induce DP properties. (**b**) Hierarchical clustering analyses indicated that hDP cells, LNGFR(+)THY-1(+) iMCs, and RA-DPAC-treated LNGFR(+)THY-1(+) iMCs (iDPSCs) possessed distinct molecular signatures. **(c)** Loss of multipotency-related and key MSC genes during RA-DPAC treatment suggested successful committed differentiation. The number in brackets indicates the number of genes in the cluster. **(d)** Intrinsic up-regulation of human DP genes in LNGFR(+)THY-1(+) iMCs delineated by cluster analysis. See also Figure S1. **(e)** Further upregulation of representative DP genes in iDPSCs following RA-DPAC treatment. **(f)** Up-regulation of DP signature genes in iDPSCs confirmed by real-time PCR. **(g)** DP-like morphology and increased alkaline phosphatase expression in iDPSCs. Scale bar = 20 μm Data were obtained with the WD39 hiPSC-line. hDPCs, human dermal papilla cells; iDPSCs, induced DP substituting cells.

Previously, we identified hDP marker genes whose expression levels were correlated with *in vivo* hair-inductive capacity^7^. By monitoring the expression levels of these genes, DP cell-activating culture (DPAC) medium containing WNT, BMP, and FGF activators was successfully developed, which restored once-impaired DP properties in serially passaged hDP genes^7^. To examine whether LNGFR(+)THY-1(+) iMCs could be programmed into dermal cells functionally analogous to hDP cells, this subpopulation was exposed to retinoic acid (RA) and subsequently to DPAC (Fig. 3a).

Microarray comparison between primary cultured hDP, LNGFR(+)THY-1(+) iMCs and RA-DPAC-treated LNGFR(+)THY-1(+) iMCs indicated that these cell populations possessed broadly distinct global gene expression profiles, as demonstrated by hierarchical clustering analyses (Fig. 3b). Previous studies have shown that simple comparison of global expression profiles is not sufficient to conclusively determine the DP properties of a tested cell population^7^,^16^. Therefore, we next evaluated changes in individual gene expression levels following RA-DPAC treatment.

After exposure to RA and DPAC, LNGFR(+)THY-1(+) iMCs showed down-regulation of multipotency-related or MSC genes, including *NANOG, ZSCAN10, FZD5, BMP7* and *ZFP64*^30^,^31^,^32^,^33^,^34^, suggesting that these cells had differentiated from their multipotent state (Fig. 3c). Interestingly, LNGFR(+)THY-1(+) iMCs intrinsically expressed hDP signature genes, including *RGS2, BMP4, LEF1* and *BAMBI*^7^, and maintained their expression levels following RA-DPAC treatment (Fig. 3d, Supplementary Fig. 2). RA-DPAC-treated LNGFR(+)THY-1(+) iMCs showed higher expression levels of additional hDP signature genes, represented by *DIO2, LPL* and *SNCAIP*^7^ than did hDP cell controls (Fig. 3e).

Microarray analysis included genes with significantly high expression levels, and therefore some key hDP genes may not have been analysed if their expression levels were below the threshold^35^. Quantitative reverse transcription polymerase chain reaction (qRT-PCR) analyses using samples obtained from different experimental batches were conducted, which indicated that key hDP genes^7^,^16^ such as *ALPL, WIF1, HEY1, LRP4, RGS2, GUCY1A3*, and *BAMBI*, were indeed up-regulated following RA-DAPC treatment in iMCs (Fig. 3f). Consistent with this observation, RA-DPAC-treated LNGFR(+)THY-1(+) iMCs gradually changed their shape and morphologically resembled cultured hDP cells with increased alkaline phosphatase activity, one of the most commonly used markers of DP function in DPAC^7^,^16^,^36^ (Fig. 3g).

These findings suggested that RA-DPAC-treated LNGFR(+)THY-1(+) iMCs, referred to as induced DP substituting cells (iDPSCs), reproduced a variety of, if not all, hDP properties and might exhibit a capacity to contribute to HF regeneration, the most characteristic property of DP cells. Recent investigations have suggested that forced cell aggregation can ameliorate DP properties in cultured hDP cells^7^,^16^. However, a pilot study demonstrated that cell aggregation was not successfully achieved in DPAC conditions, implying that additional inventions are necessary for further enhancement of DP properties in iDPSCs. Therefore, non-aggregated iDPSCs were used for downstream analyses.

### iDPSCs exhibited bi-directional epithelial-mesenchymal interactions with keratinocytes in the HF

**Figure 4:**
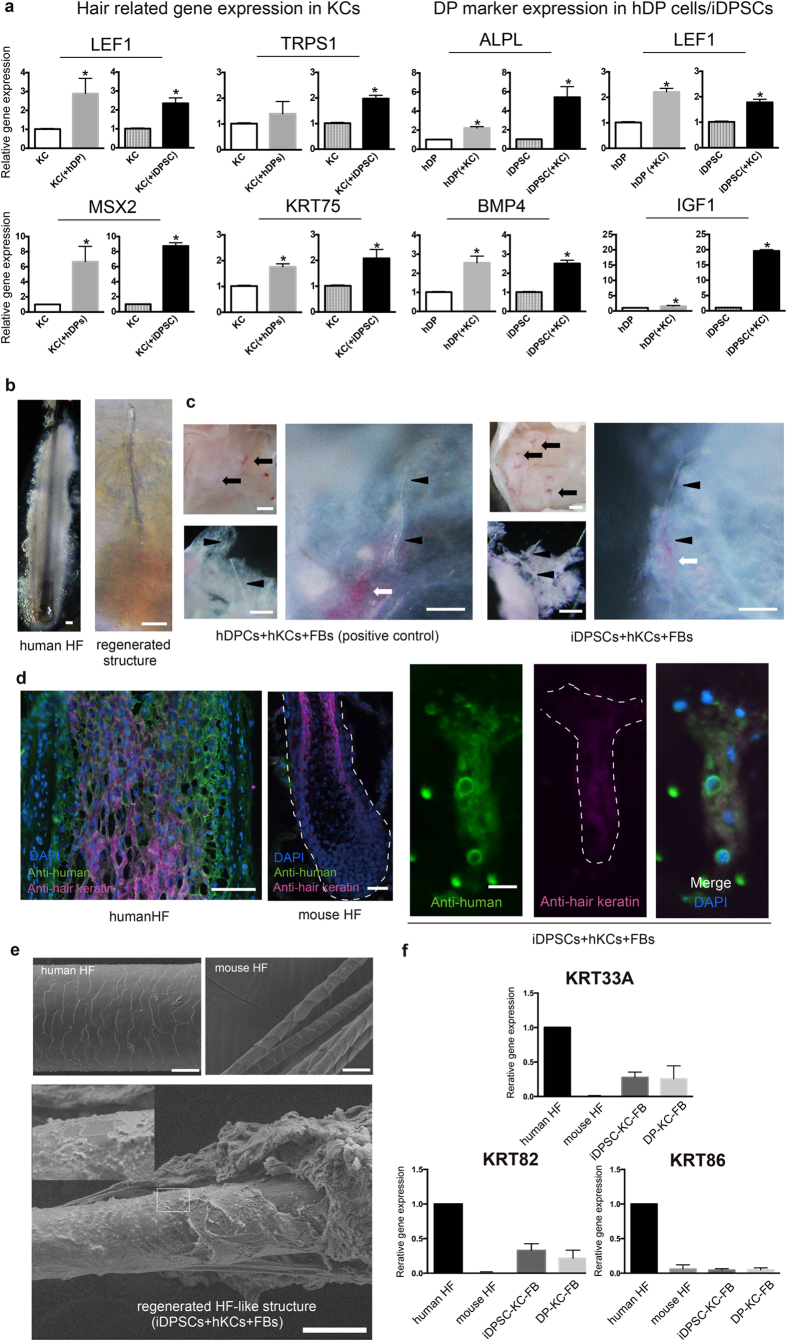
iDPSCs exhibit functional DP properties both *in vitro* and *in vivo.* **(a)** hDP cells and iDPSCs increased hair related KC gene expression in co-cultures with hKCs. At the same time, hDP cells and iDPSCs exhibited up-regulated DP biomarker genes in co-culture. (*P < 0.05). **(b)** Morphological comparison between human HF and a representative regenerated structure. **(c)** Co-grafting of hKCs and hDPCs (right) or iDPSCs (left) covered with FBs gave rise to cystic structures with focal aggregates (arrows), which contained fine HF-like structures (arrowheads), suggesting DP properties of iDPSCs. In (**b,c**), hDPCs or iDPSCs were stained red with CellBrite Orange Cytoplasmic Membrane Dye. **(d)** Double immunofluorescent staining of human and mouse HF and regenerated structures with anti-human cytoplasmic (green) and hair keratin red monoclonal antibodies. **(e)** Scanning electron microscope (SEM) images of human and mouse hair shafts and a regenerated structure. **(f)** Human hair-specific gene expression was detected in hDPC-hKC and iDPSC-hKC co-transplants. Scale bars for (**b**) = 100 μm (**c**) = 100 μm, (**d**) = 5 μm, rightmost, 20 μm (**e**) = 20 μm. Data were obtained with the WD39 hiPSC-line. hDPCs, human DP cells; hKCs, human keratinocytes; FBs, fibroblasts; HF, hair follicle. See Supplementary Figs. 2–5 for related information.

The ability to bi-directionally communicate with human keratinocytes (hKCs) to up-regulate epithelial or mesenchymal hair-related genes has been regarded as one of the most characteristic features of DP cells^7^,^37^ and a co-culture system in which hKCs and hDP cells share the same medium has been widely used as a gold standard^38^. To assess whether iDPSCs could interact with hKCs to mimic epithelial-mesenchymal interactions in HFs, this established system was adopted (Fig. 4a). Compared with expression in hKCs without co-culture used as a controls, hDP cells and hiPSC-DPSCs up-regulated hair KC-related gene in co-cultured hKCs (*LEF1, TRPS1, MSX2* and *KRT75*)^37^, indicating their capacity to communicate with hKCs (Fig. 4a). Up-regulation of most hair KC genes tended to be higher in hiPSC-DPSCs, yet the differences were not remarkable.

Bi-directional crosstalk between hDP cells/iDPSCs and hKCs was demonstrated by a reciprocal increase in the fold change in DP biomarkers (*ALPL, LEF1, BMP4* and *IGF1*) in co-cultured hDP cells/iDPSCs. Up-regulation of *ALPL* and *IGF1* was more evident in iDPSCs (P < 0.05), further supporting that iDPSCs might mimic some DP activities.

### iDPSCs contributed to formation of hair-like structures *in vivo*

In mice, co-grafting of keratinocytes and DP cells into immunodeficient mice yields complete HF structures^39^. Several studies have reported successful regeneration of HFs using human cells, however HF structures were formed when human cells were combined with mouse cells or specific human cells (e.g., neonatal cells)^13^,^40^. Accordingly, a fully stable hair reconstruction assay using widely available human epithelial and dermal cells has not been established. In fact, we attempted major HF regeneration assays represented by the patch assay using normal adult hKCs and DP cells; however, unlike hair-containing cystic structures formed by mouse cells, we could only generate barely detectable tiny structures, possibly consisting of the unabsorbed remaining human cells in the mouse *in vivo* environment (Supplementary Fig. 3). Recent studies have suggested that cell compartmentalisation can enhance epithelial-mesenchymal interactions^41^,^42^, and humanisation of the microenvironment may be beneficial for maintaining human cells in mice^43^. Taking advantage of these approaches, we developed an assay for evaluation of the *in vivo* hair inductive capacity in human cells. In this assay, human DPs, non-induced LNGFR(+)THY-1(+) iMCs or iDPSCs were combined with normal hKCs and were densely placed in a drop of collagen gel, covered with human fibroblasts (FBs) and transplanted subcutaneously into C.B-17/IcrHsd-*Prkdc*^*scid*^ mice (*n* = 28 for hDP cells, *n* = 4 for LNGFR(+)THY-1(+) iMCs and *n* = 24 for iDPSCs in seven independent experiments. (see Table 2 for summary).

The grafted composites formed cystic-like structures 5–6 weeks after transplantation irrespective of the cells grafted (Fig. 4b,c). Interestingly, when these cystic structures were carefully microdissected, fine structures resembling the hair shaft with labelled hDP cell/iDPSC cell aggregates at their roots (Fig. 4b,c) were observed in 20 of 28 hKCs/hDP/FB cell-grafted sites in 6 of 7 experiments (71.4%). Intriguingly, similar structures were detected in 7 of 20 and 1 of 4 sites where hKCs/WD39-iDPSCs/FBs and hKCs/414C2-iDPSCs were implanted, respectively (35.0% and 25.0%, respectively) in 5 of 7 experiments (Table 2). The number of regenerated structures per site was limited (approximately 2–5 per cyst). The combination of non-induced LNGFR(+)THY-1(+) iMCs with hKCs and FBs did not yield such structures (*n* = 4) (Table 2 and Supplementary Fig. 4). Control mice in which hDP cells, hKCs, non-induced LNGFR(+)THY-1(+) iMCs and iDPSCs were transplanted alone or with FBs did not give rise to HF-like structures (Table 2).

Regenerated structures were far smaller (shaft diameter <30 μm and total length ≤2 mm) than human anagen HFs (Fig. 4b,c). However, immunohistochemical examination indicated that they expressed both human cytoplasmic markers and hair keratin (Fig. 4d). In addition, scanning electron microscope (SEM) analyses of the structures regenerated from co-grafted hKCs and iDPSCs demonstrated hair shafts with flattened cuticles resembling those of human hair (Fig. 4e), although the outer root sheath was not apparent. Furthermore, subsequent qRT-PCR analysis demonstrated that up-regulation of human hair shaft genes KRT33A, 82, and 86 in the cysts formed from hKCs/hDPcells/FBs and hKCs/iDPSCs/FBs (Fig. 4f) but not in the area transplanted with hKC/iMCs coved with FBs (Supplementary Fig. 5).

These findings suggest that, similar to hDP cells, iDPSCs contribute to the formation of fibre structures with a hair cuticle-like coat mimicking the hair shaft, as a result of the interaction with hKCs.

### iDPSCs mimic the pharmacological response of DP cells to minoxidil sulfate

**Figure 5:**
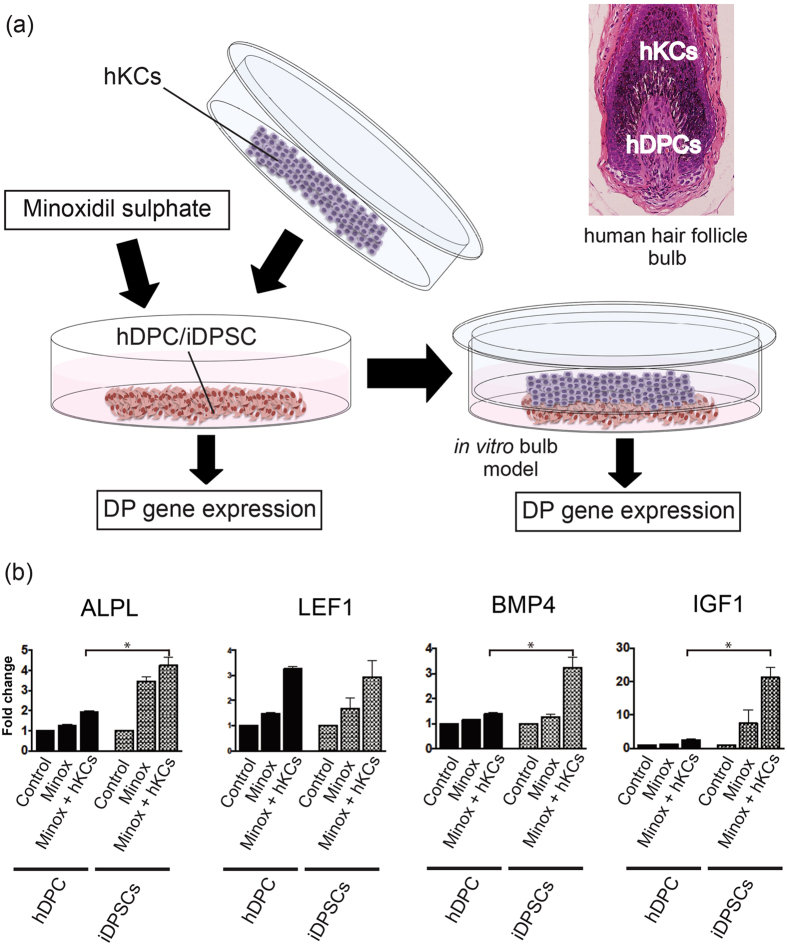
iDPSCs mimic the pharmacological response of DP cells to minoxidil sulfate. (**a**) Schematic illustrating the experimental procedure and a pathological image of human hair follicle bulb. A co-culture model reproduced the anatomical relationships between hKCs and hDPCs *in vivo*. **(b)** Effects of minoxidil sulfate on DP signature gene expression in cultured hDPCs or iDPSCs with or without hKC co-culture. Note that up-regulation of all DP genes tested except for *LEF1* was more remarkable in iDPSCs than in hDPCs in the presence of hKCs and minoxidil sulfate (*P < 0.05). Minox, minoxidil sulfate. Data were obtained using the 414C2 hiPSC-line, because this line survived better than did the WD39-hiPSC line in co-culture. hDPCs, human DP cells; hKCs, human keratinocytes.

Minoxidil is a clinically used hair growth promoter that enhances hair KC proliferation and activates hDP cells to induce growth factors^44^. IGF-1 is among these growth factors, and has been shown to exhibit a potent hair elongation effect^45^,^46^,^47^,^48^. To examine whether iDPSCs would be useful for future drug discovery for hair diseases, their pharmacological response to minoxidil was compared with that of hDP cells (Fig. 5a). Addition of minoxidil sulfate enhanced the expression of DP marker genes *ALPL* and *IGF1* in iDPSCs more intensely than in hDP cells, while *LEF1* and *BMP4*^7^,^14^,^36^ were moderately up-regulated in both populations (Fig. 5b). When minoxidil sulfate was added to hKCs-hDP cells or hKC-iDPSC co-cultures mimicking the HF bulb (Fig. 5a), iDPSCs showed stronger up-regulation of *ALPL, BMP4* and *IGF1* than did hDP cells (P < 0.05) (Fig. 5b).

These observations indicate that iDPSCs reproduce some aspects of pharmacological responses of hDP cells, which could be potentiated in the presence of hKCs, suggesting that iDPSCs may serve as useful tools for the discovery of new reagents to promote hair growth.

## Discussion

Preparation of sufficient quantities of functional DP cells is essential to achieve successful human HF bioengineering^8^,^9^. Previous studies have demonstrated that DP properties, including the hair-inductive capacity, were significantly impaired following *in vitro* expansion in the case of hDP cells^7^,^16^,^49^. Although functional restoration of cultured hDP cells is possible to some extent, current technology is insufficient to allow complete restoration^7^,^16^. Limitations in sample supply and the laborious manual microdissection required to isolate hDP cells have led to a clear demand for alternative approaches to prepare functional DP equivalents^7^,^16^.

Recent studies reported successful generation of mesenchymal cells with plasticity from hiPSCs^50^,^51^, suggesting that DP cells or their equivalents may be generated from hiPSCs via differentiation to mesenchymal cells. In this study, hiPSCs were successfully programmed into hBMSC-like mesenchymal cells, as demonstrated by the expression of fibroblastic mesenchymal cell markers including THY-1, CD166, CD44 and integrin β1^19^,^29^ and the outcome of *in vitro* differentiation assays. Additional studies aiming to enhance the potential of future applications of iMCs would include a more precise biological definition (including ‘stemness’) of iMCs by further characterisation at the single-cell level and *in vivo* differentiation assays^18^.

A recent study demonstrated that reasonably pure proliferative and multipotent cell populations could be isolated from human bone marrow cells using cell surface markers LNGFR and THY-1^19^. In comparison of the three up-regulated mesenchymal lineage signature genes, LNGFR(+)THY-1(+) iMCs might be less multipotent than LNGFR(+)THY-1(+) BMSCs. However, as the LNGFR(−)THY-1(+) cell population could not be propagated well *in vitro* and died out during differentiation, it would be reasonable to select and use LNGFR(+)THY-1(+) iMCs for DP property induction.

Global gene expression analyses suggest that iDPSCs partially reproduce the molecular signature of DP cells. iDPSCs likely represent a heterogeneous population, and it is therefore feasible that some unidentified specific subsets possess DP cell activity. Preferential DP cell surface markers have not been identified in hDP cells, unlike murine DP cells^7^. Accordingly, iDPSCs could not be further selected. Incomplete conversion may also be attributed to the use of DPAC. DPAC is a defined condition and its effects on dermal cells have been well-characterised^7^, allowing systematic evaluation of biological alterations in DPAC-exposed cell populations. However, DPAC activates WNT, BMP and FGF pathways but not others, including SHH and NOTCH, which are crucial for hDP property maintenance^52^,^53^. Further modification of the differentiation protocol may therefore be necessary to more accurately assess whether LNGFR(+)THY-1(+) iMCs can be programmed into hDP cells.

Our induction protocol appeared to elicit some functional DP properties in LNGFR(+)THY-1(+) iMCs, as demonstrated by a co-culture experiment and an *in vivo* hair induction assay. For better characterization, hiPSC or other comparisons needs to be co-cultured with hKCs to assess if up-regulation of HF-related genes were specifically observed with hDP cells or iDPSCs. However, the detection of increase in gene expression requires the use of DMEM: F12 without supplements. Probably because of hKC derived-factors^54^, hDP and iDPSCs could survive during an incubation period. As the co-culture model adopted in this study has been widely used as a readout of DP properties^7^,^37^,^38^, the resultant up-regulation in HF-related genes in both epithelial and mesenchymal components supported that iDPSCs recapitulated *in vitro* DP properties at least to some extent.

Ideally, the hair inductive capacity needs to be assessed by standard hair reconstitution assays, including the chamber or patch assays which have been used to demonstrate the hair inductive capacities of mouse and canine cells^55^,^56^. Recent investigations indicated that, in the case of human cells, the hair regeneration efficiency is also markedly affected by the biological properties of both keratinocytes and DP cells^13^,^57^. Despite many attempts using these approaches, HF-like structures were not observed after co-grafting normal adult hKCs and hDPs, prompting us to develop an alternative assay.

Taking advantage of cell compartmentalisation and humanisation of the microenvironment^41^,^42^,^43^, we managed to establish an approach by which hair shaft-like structures were generated using normal adult hKCs and hDP cells. Even using this approach, incomplete and fine hair shaft-like structures were obtained from a positive control. We are aware that the assay requires further refinements, because regenerated structures were not always formed and those formed were very small and of incomplete HF morphology. Based on the past obsevations, this is not unusual with HF regeneration attempted exclusively with cells of human adult origin^7^,^16^,^57^. The size and rarity made it technically challenging to analyse regenerated structures further. However, immunohistochemical examination and SEM analysis respectively detected human and HF-specific markers and hair shaft cuticle-like structures in HF-like structures. In addition, marked up-regulation of human hair keratin genes were only observed in a tissue formed in the presence of hDP cells or iDPSCs but not iMCs. These findings, together with their capacity to communicate with hKCs to up-regulate HF genes *in vitro*, suggested that it would be reasonable to conclude that iDPSCs mimic some properties of hDP cells.

Theoretically, the intensity of DP gene expression levels in iDPSCs needs to be compared with those in freshly isolated hDP cells. Yet, hDP cell isolation still largely depends on mechanical microdissection in which contamination of hair matrix keratinocytes and melanocytes is inevitable^7^. This makes direct comparison of the data difficult. In the present study, iDPSCs showed stronger expression of some DP markers than did cultured hDP cells but less frequent induction of hair shaft-like structures *in vivo*. This discrepancy may have been due to gradual loss of DP properties in the *in vivo* environment due to the absence of DPAC activation. Local introduction of DPAC factors into the transplantation site could ameliorate the trichogenic activity of iDPSCs.

Although hDPs and iDPSCs were condensed at the root of regenerated structures, they did not form distinct cell aggregates, as observed with *in vivo* hDPs. It has been reported that cell aggregation partially restores DP cell properties^7^,^16^,^40^,^58^. It is possible that forced cell aggregation to mimic DP morphology prior to co-grafting with hKCs may have enhanced the inductive capacity of iDPSCs. However, this approach remains challenging, because it is difficult to form cell aggregates in DPAC conditions. This possibility should be examined in future studies.

We are aware that how iDPSCs mimic biological behaviors of *bona fide* hDP cells, especially in terms of trichogenic activity, were insufficiently evaluated in this study mainly because of technical limitations described above. The possibility that iDPSCs might belong to another mesenchymal lineage but happened to exhibit some DP cell properties cannot be totally ruled out. Further characterization of iDPSCs, especially focusing on their hair inductive capacity, using high-resolution techniques is necessary to definitively conclude their cell types. Still, induction of the cells capable of interacting with hKCs in the context of HF biology using hiPSCs would be beneficial to applications for which complete reproduction of DP cell properties may not always be indispensable.

For instance, the advantage of using iDPSCs for pharmaceutical screening was supported by the observation that iDPSCs showed up-regulation of DP markers and IGF-1, a major mediator involved in hair growth promotion^59^, in response to minoxidil sulfate in co-culture with hKCs. Preparation of a co-culture system using hiPSC-derived KCs and iDPSCs derived from individuals genetically predisposed to hair loss disease, including androgenetic alopecia and female pattern baldness, may allow for more specific and effective drug discovery for these conditions^9^,^21^.

Another potential future improvement to the current approach to generate DP or equivalent cells from hiPSCs would be generation of hiPSC-derived neural crest cells^60^. DP cells in the craniofacial area originate from neural crest cells^61^,^62^ and a protocol for the differentiation of neural crest cells from human pluripotent stem cells has been established^63^, it would be more efficacious to use hiPSC-derived neural crest cells instead of iMCs.

In summary, the present study describes a protocol to generate LNGFR(+)THY-1(+) iMCs and induce, at least in part, representative DP cell properties in this cell population. With further improvement, iDPSCs may serve as useful tools for HF bioengineering and facilitate the discovery of novel drugs for the treatment of scalp and hair diseases. To expand the range of applications, in-depth dissection of the molecular machinery of HF morphogenesis is required to increase the efficiency of the induction protocol.

## Methods

### Preparation of hiPSCs and hDP cells

Established hiPSC lines generated by introduction of reprogramming factors into dermal fibroblasts by retroviruses (201B7 25 and WD39^26^) or episomal plasmid vectors (414C2^27^) were maintained as described previously^37^. Induction of iMCs was commenced when passage 20–35 hiPSC lines were 80–90% confluent. hDP cells were prepared from human scalp samples obtained during surgery^7^. All experimental procedures were approved by the Institutional Review Board of Keio University (Protocol No. 2005–0075) and performed in accordance with the university’s ethical guidelines. All human donors provided written informed consent in accordance with the Declaration of Helsinki.

### Generation of iMCs

EBs were formed as reported previously^37^ and kept for 2 days floating in hiPSC medium without FGF2. Subsequently, EBs were cultured in Stempro MSC-SFM CTS (Life Technologies) on a humanised substrate (CELLstart CTS; Life Technologies) and cultured for a further 9–11 days until confluent. Resultant iMCs were collected with TrypLE Express (Life Technologies) and seeded at a density of 1 × 10^5^ cells/cm^2^. The detailed protocol is provided in Supplementary Materials and Methods.

### Flow cytometric analysis

iMCs (passage 3) and human bone marrow cells were incubated with mouse anti-human CD29, CD44, CD90, CD166, and CD45 monoclonal antibodies conjugated with phycoerythrin, allophycocyanin, or fluorescein isothiocyanate (Biolegend) and isotype controls at 1:20 dilution for 30 min. Cells were analysed on a BD FACSCanto II using BD FACSDiva software (BD Biosciences). The data were analysed with Flowjo software (Tree Star).

### Differentiation of iMCs into three mesenchymal lineages

iMCs were expanded to 70–80% confluence and cultured in osteogenic, adipogenic and chondrogenic induction media (Lonza, Walkersville, MD). Successful differentiation was confirmed after 4–5 weeks by staining with Alzarin red (Millipore), Oil red O (Muto Pure chemicals) and Toluidine blue (Wako).

### Sorting for LNGFR(+)THY-1(+) iMCs

iMCs were detached, dissociated and incubated with mouse anti-human CD271 phycoerythrin-conjugated (Biolegend) and anti-human CD90 allophycocyanin-conjugated monoclonal antibodies (Biolegend) for 30 min. Matched isotype controls are used as negative controls. Flow cytometric sorting was then performed on a MoFlo XDP (Beckman Coulter)^19^.

### DP property induction

When sorted LNGFR(+)THY-1(+) iMCs reached 80–90% confluence, DP induction was started using Dulbecco’s Modified Eagle’s Medium (DMEM) supplemented with 10% foetal bovine serum (FBS) and 0.01 mM all-*trans* retinoic acid (Sigma) (days 0–4). On day 4, induction medium was then changed to DMEM containing 10% FBS, 20 ng/ml bFGF (Peprotech), 200 ng/ml human recombinant BMP2 (R&D Systems, Minneapolis, MN, USA), and 1 μM 6-bromoindirubin-3′-oxime (Sigma), an inhibitor of GSK-3α/β in the WNT signalling pathway (days 4–9).

### Quantitative reverse transcription polymerase chain reaction

Quantitative reverse transcription polymerase chain reaction analysis was performed as described previously^7^,^37^ using an Applied Biosystems StepOnePlus Real-Time PCR system (Life Technologies). Primers are listed in Supplementary Table S1.

### Microarray analyses

Total RNA was isolated from two sets of primary cultured hDP cells, LNGFR(+)THY-1(+) iMCs and iDPSCs. Cyanine-3-labeled cRNA was prepared with the Low Input Quick Amp Labeling kit, One-Color (Agilent), hybridised to SurePrint G3 Human Gene Expression 8 × 60 K v2 (Agilent), and scanned according to the manufacturer’s protocol. The expression data were normalised and clustered by both unsupervised hierarchical and k-means (50 clusters) clustering methods using GeneSpring GX software with default parameters (Agilent).

### Co-culture of hDPCs /iDPSCs with hKCs

hDP cells/iDPSCs cells were co-cultured with 2.5 × 10^5^/cm^3^ hKCs (CELLnTEC advanced cell systems, Bern, Switzerland) seeded onto overlying collagen coated permeable Transwell inserts (Corning, Corning, NY, USA) in DMEM:F12 with or without 10 μM minoxidil sulphate (Sigma). As controls, hDP cells, iDPSCs and hKCs were cultured individually in DMEM:F12. After 4 days total RNA was extracted for real-time PCR analyses. See Supplementary Materials and Methods for details.

### *In vivo* hair induction assay

hDP cells (passage 2 or 3; average 2.6 × 10^5^), iMCs or iDPSCs (average 3.6 × 10^5^) were stained with CellBrite Orange Cytoplasmic Membrane Dye (Biotium), mixed with Matrigel Matrix Growth Factor Reduced (BD Biosciences) and placed onto thin silicone sheets. Subsequently, cultured human adult KCs (average 1.5 × 10^5^) in Matrigel were placed on top. Then, composites were fully covered with cultured human fibroblasts (1.2 × 10^4^/μl) in Matrigel. Final composites were transplanted into the dorsal region of anesthetised 8-week-old female C.B-17/IcrHsd-*Prkdc*^*scid*^ mice (Japan SLC). After 5–6 weeks, grafts were harvested and microdissected using watchmaker’s forceps and fine needles under light microscopy. All animal procedures were performed in accordance with the guidelines of the Science Council of Japan and approved by the Keio University Institutional Animal Care and Use Committee.

### Immunohistochemistry

Microdissected tissue was embedded in OCT compound (Sakura Finetek) and sectioned. Sections were incubated with anti-human cytoplasm antibody (1:00, STEM121; StemCells), followed by Alexa Fluor 488 goat anti-mouse IgG (H+L) antibody (Life Technologies), washed and subsequently stained with mouse anti-hair cortex cytokeratin antibody (AE13; Abcam, Cambridge, UK) conjugated with Alexa Fluor 647 using a Zenon Mouse IgG1 Labeling kit (Life Technologies). Details are described in the Supplementary Materials and Methods.

### Scanning electron microscopy

Microdissected samples were prefixed with 2.5% glutaraldehyde/30 mM HEPES, pH 7.4 (TAAB Laboratories Equipment) at 4 °C for 2 h and postfixed with 1% OsO_4_/30 mM HEPES, pH 7.4 (TAAB Laboratories Equipment Ltd.) at room temperature for 1 h. After dehydration, conductive staining was performed using 10% phosphotungstic acid/100% ethanol. Samples were subjected to scanning electron microscope investigation using an SU6600 low-vacuum electron microscope (Hitachi High-Tech) with an acceleration voltage of 7 kV, working distance of 5 mm and vacuum condition of 50 Pa, using an environmental secondary electron detector.

### Assessment of the effect of minoxidil on hDPs/iDPSCs

hDP cells/iDPSCs were cultured with or without 10 μM minoxidil sulfate (Sigma) as describe above. After 4 days, total RNA was extracted for real-time PCR analyses.

### Statistical analysis

The statistical significance of differences in results from real-time PCR analysis was determined using the two-sided Student’s t-test with P < 0.05 taken to indicate significance. Error bars provided in the figures represent standard error of the mean (SEM).

### Accession number

The Gene Expression Omnibus accession number for the microarray reported in this paper is GSE61511.

## Additional Information

**How to cite this article**: Veraitch, O. *et al*. Induction of hair follicle dermal papilla cell properties in human induced pluripotent stem cell-derived multipotent LNGFR(+)THY-1(+) mesenchymal cells. *Sci. Rep.* **7**, 42777; doi: 10.1038/srep42777 (2017).

**Publisher's note:** Springer Nature remains neutral with regard to jurisdictional claims in published maps and institutional affiliations.

## Supplementary Material

Supplementary Information

## Figures and Tables

**Table 1:** Summary of mesenchymal lineage marker expression and induction efficiency of each iPSC lines.

Cell line	Positive surface markers	Osteoblasts	Adipocytes	Chondrocytes	% LT*** positive cells
WD39	CD29, 44, 90, 166	+++	+	++	11.66 ± 1.72%
201B7	CD29, 44, 90, 166	++	+	++	6.40% ± 2.97%
414C2	CD29, 44**, 90, 166	++	+	+	14.52% ± 2.06%
hBMSCs*	CD29, 44, 90, 166	+++	+++	+++	0.05% ± 0.01%

Positively stained cells for each lineage: +++: 80%<, ++: 20–79%, +: 1–19%,

*hBMSCs: human bone marrow stromal cells; **moderate expression; ***LT: LNGFR/THY-1.

**Table 2:** Summary of co-transplantation experiments.

Transplanted cells	Grafted sites	Sites with hair-like structures
hKCs + FBs + hDPs	28	20
hKCs + FBs + iDPSCs (WD39)	20	7
hKCs + FBs + iDPSCs (414C2)	4	1
hKCs + FBs + iMCs (WD39)*	4	0
FBs + hDPs	8	0
FBs + iDPSCs (WD39)	6	0
FBs + iDPSCs (414C2)	2	0
hKCs + FBs	6	0
hKCs + hDP	2	2
hKCs + iDPSCs (WD39)	2	0
hDPs	3	0
iDPSCs (WD39)	3	0
hKCs	1	0
FBs	3	0

*Non-DPAC treated LNGFR(+)THY-1(+) iMCs.
