# Supplementary material for: Induction of hair follicle dermal papilla cell properties in human induced pluripotent stem cell-derived multipotent LNGFR(+)THY-1(+) mesenchymal cells
Source: Sci Rep. 2017 Feb 21;7:42777. doi: 10.1038/srep42777 (PMC5318903; doi:10.1038/srep42777)
Supplement: Supplementary Information [file srep42777-s1.pdf]

Supplementary information for

Scientific Reports Article

Induction of hair follicle dermal papilla cell properties in human induced pluripotent stem cell-derived multipotent LNGFR(+)THY-1(+) mesenchymal cells

Ophelia K. Veraitch[1], Yo Mabuchi[2,3], Yumi Matsuzaki[2,4], Takashi Sasaki[5], Hironobu Okuno[2] , Aki Tsukashima[1,6], Masayuki Amagai[1], Hideyuki Okano[2*] and Manabu Ohyama[1,6*]

Departments of [1]Dermatology and [2]Physiology, Keio University School of Medicine
35 Shinanomachi, Shinjuku-ku, Tokyo, 160-8582 JAPAN
[3]Department of Biochemistry and Biophysics, Graduate School of Health Care Sciences, Tokyo Medical and Dental University, 1-5-45 Yushima, Bunkyo-ku, Tokyo, 113-8510, JAPAN
[4]Laboratory of Tumor Biology, Department of Life Sciences, Faculty of Medicine, Shimane University, Shiojicho 89-1, Izumo-shi, Shimane, 693-8501, JAPAN
[5]KOSÉ Endowed Program for Skin Care and Allergy Prevention, Keio University School of Medicine, 35 Shinanomachi, Shinjuku-ku, Tokyo, 160-8582 JAPAN
[6]Department of Dermatology, Kyorin University School of Medicine,
6-20-2 Shinkawa, Mitaka-shi, Tokyo, JAPAN

*Corresponding authors

Supplementary materials and methods

Information on hiPSC lines

Three established hiPSC cell lines were used for mesenchymal cell (iMC) induction. 201B7 and WD39 hiPSC lines (Takahashi et al, 2007; Imaizumi et al, 2012) were obtained from dermal fibroblasts derived from facial and scalp dermis respectively and generated by retroviral introduction of *POU5F1, SOX2, KLF and MYC*. 414C2 hiPSC line was generated by introduction of *OCT3/4*, *SOX2*, *KLF4*, *L-MYC*, *LIN28* and *GLIS1* to dermal fibroblasts using episomal plasmid vectors (Okita et al, 2012). 414C2 hiPSC line can be utilized under a material transfer agreement contract with the Center for iPS Research and Application, Kyoto University (director, Prof. Shinya Yamanaka).

Generation of iMCs

To induce MC differentiation, passage 20-35 healthy hiPSC lines (Hussein et al, 2011) were used. When hiPSC colonies had reached 80 - 90% confluence embryoid bodies (EBs) were formed to differentiate hiPSCs by loosing the attachment of feeder cells. Prior to making EBs, all hiPSC colonies with signs of differentiation were manually removed. hiPSC clumps were then enzymatically detached from feeders using CTK solution containing 0.25% trypsin, 20% KSR, 0.1 mg/ml collagenase IV (Life Technologies), 1 mM calcium chloride (Sigma) and 1X PBS (Sigma). After 5 minutes incubation with CTK solution 10 mls of hiPSC medium was added. The cells were then gently transferred to a 15ml tube and centrifuged at 300 rpm for 3 minutes at room temperature. Use of 5 ml

pipette (Greiner bio one, Kremsmünster, Austria) for handling EBs would minimize the risk of cell clump breakdown at the steps hereafter. The cells were re-suspended in 10 ml of hiPSC medium and seeded onto gelatin-coated dishes for 90 minutes at 37^0C, 3% CO_2 allowing removal of remaining feeder cells. hiPSC colonies were then gently collected, centrifuged at 300 rpm for 3 minutes at room temperature and transferred into floating culture dishes (Superior Petri Dishes; Kord-Valmark, Ontario, Canada) filled with hiPSC medium without FGF2 and kept at 37^0C and 3% CO_2. A confluent 100 mm hiPSC dish generated 1 x 100 mm dish of floating EBs.

After 2 days in floating culture, resultant EBs were collected and allowed to settle by gravity. Supernatant was discarded and each 100 mm dish of EBs were re-suspended in 10 mls of Stempro MSC-SFM CTS (Life Technologies) and transferred into a 75 cm^3 flask. Culture flasks had been coated with CELLstart CTS (Life Technologies) 1:100 in PBS with calcium and magnesium (Life Technologies) at 85 µl/cm^3 overnight at 4^0C or 1 hour at 37^0C, and excess coating suctioned before EBs suspended in Stempro MSC-SFM CTS were seeded and cultured overnight at 37^0C, 3% CO_2. The following day, non-attached EBs were removed by gently washing culture flasks with PBS twice, before adding fresh culture medium. Thereafter, two PBS washes followed by fresh culture medium addition was performed every 2 days.

When confluent (day 11-13), iMCs derived from EBs were passaged. iMCs were washed with PBS and 5 mls of TrypLE Express (Life Technologies) added to each flask of iMCs and incubated for 5 minutes at 37^0C, 5% CO_2. After checking iMC detachment by agitating dishes, iMCs were collected with ice cold PBS and centrifuged at 1200 rpm at room temperature for 5 minutes. iMCs were passed through a 100 µl cell strainer and

seeded at a density of 1 x 10^5 cells / cm ^{2} in Stempro MSC-SFM CTS on CELLstart CTS coated dishes, with medium changes on alternate days until confluent and ready for passaging.

Flow cytometric analysis

For positive controls, bone marrow-derived cells at P2-P5 were used (Mabuchi et al, 2013). hMSC medium consisted of low glucose DMEM (Nacalai tesque, Kyoto, Japan) supplemented with 20% FBS (Hyclone, Logan, UT), 20 ng/ml bFGF (Peprotech, Rocky Hill, NJ), 10mM HEPES (Nacali tesque) and 1 % penicillin and streptomycin (Mabuchi *et al*., 2013) were used for their maintenance. iMCs and control cells were detached with pre-warmed cell dissociation buffer enzyme free Hanks' based (Life Technologies) and collected with FACS buffer (ice cold PBS containing 5% fetal bovine serum). After washing, cells were incubated with primary antibodies at 1:20 dilution for 30 minutes on ice and in the dark; mouse anti-human THY-1 (CD90; allophycocyanin [APC]-conjugated, Cat. No. 328114), CD166 (phycoerythrin [PE]-conjugated, Cat. No.343903), CD44 (PE-conjugated, Cat. No.338807), CD29 (PE-conjugated, Cat. No.303003), HLA-DR (APC-conjugated, Cat. No.307610), CD45 (fluorescein isothiocyanate [FITC], Cat. No. 304005) and CD31 (PE-conjugated, Cat. No.303105) antibodies (all BioLegend, San Diego, USA). Isotype controls with matching Ig subclass and conjugates were used as negative controls. Propidium iodide was used to exclude dead cells from analysis. Cells were then analyzed on BD FACSCanto II by use of BD

FACSDiva software (BD Biosciences). The data analyses were performed with Flowjo software (Tree star, Inc., Ashland, OR).

Differentiation of iMCs to osteoblasts, adipocytes and chondrocytes

To induce osteoblast differentiation, iMCs at P1-P3 at 70-80% confluency were cultured in pre-warmed osteogenic induction medium (Lonza, Walkersville, USA) and then incubated at 37^0C and 5% CO_2. Induction medium was changed every three to four days for approximately four to five weeks. To confirm successful induction into osteoblasts, cells were fixed with 4% paraformaldehyde for 10 minutes, washed three times with PBS for 5 minutes, incubated with alizarin red (Millipore) for 20 minutes, washed with distilled water for 5 minutes and then kept in 100% ethanol at 4^0C.

To induce adipogenic differentiation, iMCs at P1-P3 at 70-80% confluency were cultured in adipogenic induction medium for 4 days and maintenance medium for 3 days (both Lonza) at 37^0C and 5% CO_2. Four to 5 cycles of this series was sufficient to induce adipocyte differentiation. To confirm successful induction into adipocytes, cells were fixed with 4% paraformaldehyde for 10 minutes, washed three times with PBS for 5 minutes, incubated with 60% 2-propanolol (diluted with distilled water) for 5 minutes, incubated with pre-filtered Oil red O (3:2 dilution with distilled water) (Muto Pure Chemicals, Tokyo, Japan) for 30 minutes, washed with 60% 2-propanolol for 1 minute, washed with distilled water for 5 minutes and kept in distilled water at 4^0C.

To induce chondrogenic differentiation, 1-2 $\times$ 10^6 iMCs at passage 1-3 were transferred into 15ml conical tube and washed in pre-warmed chondrogenic medium

(Lonza). The cells in tubes were centrifuged at 160g for 4 min at 25 °C. Subsequently, supernatant was discarded and the cells were re-suspended in 1 ml of differentiation basal medium chondrogenic medium (Lonza) supplemented with 10 ng/ml TGFβ3 (Lonza) and 500 ng/ml BMP6 (R&D Systems, Minneeapolis, MN, USA) followed by centrifugation at 160g for 4 min at 25 °C and incubation at 37^0C and 5% CO_2. Medium was changed every 3 days for 4-5 weeks. To confirm successful induction into chondrocytes, cell aggregates were fixed with 4% paraformaldehyde and paraffin-embedded and sectioned (Cut 6-µm-thick paraffin sections). The sections were deparaffinized and hydrated in distilled water. Then, the sections were stained in Toluidine blue working solution (Wako, Osaka, Japan) for 30 min, dehydrated quickly once with 95% (vol/vol) alcohol and twice with 100% ethanol. After washing in xylene three times, 10 min each, the sections were cover-slipped and kept in Entellan medium.

Flow cytometric sorting for LNGFR(+)THY-1(+) iMCs

iMC were detached with pre-warmed cell dissociation buffer enzyme free Hanks' based (Life Technologies) and collected with FACS buffer. After washing, cells were suspended in 3 mls of FACS buffer and incubated with 70 µl and 28 µl of anti-human LNGFR (PE-conjugated, Cat. No. 345106, Biolegend) and THY-1 (APC-conjugated, as described above) monoclonal antibodies for 30 minutes on ice and in the dark. Isotype controls with matching Ig subclass and conjugates were used as negative controls. Propidium iodide was used to exclude dead cells from sorting. Flow cytometric analysis of controls determined the setting for gating and sorting of LNGFR(+)THY-1(+) iMCs

fractions was performed on MoFlo XDP (Beckman Coulter, Brea, CA) as previously described (Mabuchi et al, 2013). Sorted cells were washed in DMEM containing 10% FBS three times and seeded in Stempro MSC SFM CTS on CELLStart CTS (Life Technologies) at a density of $1x10^5$ cells / cm^3 in 24 well plates. Further passaging and expansion was also performed using plastic non-coated dishes using hMSC medium.

Dermal papilla property induction

When primary sorted LNGFR(+)THY-1(+) iMCs were 80-90% confluent, dermal papilla substituting cell (DPSC) induction was started for day 0-4 using DMEM supplemented with 10% FBS and 0.01 mM all-trans retinoic acid (Sigma), changing induction medium on day 2. On day 4 of induction, medium was changed to DMEM supplemented with 10% FBS, 1 μM 6-bromoindirubin-3'-oxime (Sigma) (an inhibitor of GSK-3α/β in the Wnt signaling pathway), 200 ng/ml human recombinant BMP2 (R&D Systems, Minneapolis, MN) and 20 ng/ml bFGF (Peprotech). Medium was changed on day 6 and 8 of induction, and on day 9 total RNA was extracted for analysis of human dermal papilla signature genes.

Microarray analysis

Total RNA of cultured or sorted cells was extracted with RNeasy Mini kit (Qiagen). RNA Integrity Number (RIN) of the extracted total RNA samples was measured with 2100 Bioanalyzer (Agilent Technology, Santa Clara, CA) and we confirmed that RIN

value of all samples were more than 9.8. Cyanine-3 labelled cRNA was generated from 100 ng of total RNA with Low Input Quick Amp Labelling Kit one-color (Agilent). These labeled cRNA samples were hybridized to slide glass of SurePrint G3 Human Gene Expression 8x60K v2 microarray (Agilent) and hybridized slide glass was scanned with High-Resolution Microarray Scanner (Agilent) according to the manufacture's protocols. These scanned data were analysed by Feature Extraction software 9.1 (Agilent) to extract fluorescence intensity data for each probe. The extracted intensity data was normalized by 75th percentile methods with Gene Springs GX (Agilent) and low expression probes which intensity are less than 100 in all six samples were removed. These fluorescence intensity data were clustered by both unsupervised hierarchical and k-means (n=50) clustering methods with default setting of GeneSpring GX software. The raw microarray data files are accessible at NCBI's Gene Expression Omnibus through GEO Series accession number GSE61511 (http://www.ncbi.nlm.nih.gov/geo/query/acc.cgi?acc5GSE61511).

Quantitative Reverse Transcription-Polymerase Chain Reaction

RNA purification, cDNA generation and real-time PCR analyses were performed as previously described (Veraitch *et al*., 2013). The results are presented as mean ± standard error of means (SEMs). Statistical significance of the differences in real-time PCR analysis was determined using a two-sided Student's t test with $P<0.05$ considered significant. The data was obtained from three respective experiments individually performed in duplicate.

Co-culture of hDP cells/ iDPSCs with normal human keratinocytes

Following DP induction, iDPSCs were co-cultured with normal human keratinocytes (hKCs). Approximately 2.5 x 10^5 passage 3 hKCs (CELLnTEC advanced cell systems, Bern, Switzerland) that had been expanded and cultured in CnT-57 (CELLnTEC) were seeded onto collagen coated permeable transwell inserts (Corning, Corning, NY) in DMEM: F12 (3:1) at 37℃, 5% CO_2. For positive controls cultured hDP cells (passage 0-1) originally microdissected from human scalp samples and cultured in Follicle Dermal Papilla Growth Medium (Promocell, Heidleberg, Germany) were also co-cultured with hKCs. In addition, hDP cells/iDPSCs were co-cultured with hKCs and 10 μM minoxidil sulphate. As controls, hDP cells/iDPSCs and hKCs were cultured with minoxidil sulphate (Sigma) without co-culture for 4 days. For these cultures, medium was changed on day 2. On day 4, total RNA was extracted for real-time PCR as described above.

In vivo hair induction assay

In vivo hair induction assay was performed as previously reported by Nakao et al. (Nakao et al, 2007) with modifications. Briefly, cultured human DP cells (passage 2 or 3; average 2.6×10^5), LNGFR(+)THY-1(+) iMCs or iDPSCs (average 3.6×10^5) respectively stained with CellBrite Orange Cytoplasmic Membrane Dye (Biotium, Hayward, CA) were injected into a drop of Matrigel (BD Biosciences, Franklin Lakes, NJ) placed on thin silicone sheets (approximately 5mm × 5mm) using micropipette. Subsequently, cultured hKCs (average1.5×10^5) were injected onto human DP cell, iMC or iDPSC cell aggregates

within Matrigel drops. Then, Matrigel drops were fully covered with Matrigel containing cultured human fibroblasts (1.2×10^4/ µl). The composites were stabilized in 1:1 mixture of Dulbecco's Modified Eagle Medium (DMEM) (Sigma, St. Louis, MO) containing 10% fetal bovine serum (FBS) (Sigma) and CnT-57 medium (CELLnTEC advanced cell systems, Bern, Switzerland) for 1hour at 37^0C, 5% CO_2. Afterwards, the stabilized composites were subcutaneously transplanted into the dorsal aspect of anesthetized 8-week-old female C.B-17/IcrHsd-*Prkdc*^{scid} mice (Japan SLC, Hamamatsu, Japan). After 5-6 weeks, the grafts were harvested and microdissected using watchmaker's forceps and fine needles under a stereo high-resolution dissecting microscope (SZX16, Olympus Corporation, Tokyo, Japan).

Immunohistochemical staining

Microdissected tissue was embedded in OCT compound (Sakura Finetek, Tokyo, Japan), sectioned and stored at -80°C until used. Frozen sections were defrosted, fixed with acetone at -20°C for 10 minutes and washed with PBS for 5 minutes for three times. The sections were incubated with anti-human cytoplasm antibody (1:00, STEM121, StemCells, Newark, CA) for 2 hours at room temperature, washed and then incubated with Alexa Flour 488 goat anti mouse IgG (H+L) antibody (Life Technologies) for 1 hour at room temperature. After washing, the sections were stained with mouse anti-hair cortex cytokeratin antibody (AE13, abcam, Cambridge, U.K.) conjugated with Alexa Fluor 647 using Zenon Mouse IgG1 labeling kit following the manufacturer's protocol (Life Tchnologies) for 1 hour at room temperature. Subsequently, they were fixed with 4% paraformaldehyde for 15minutes, washed, additionally stained with Hoechst33342

(Life Technologies) and mounted with CC/mount (Diagnostic BioSystems, Pleasanton, CA). The images were captured using AxioVision (Zeiss, Oberkochen, Germany).

Scanning electron microscopy

Microdissected samples were prefixed with 2.5% gutaraldehyde/ 30mM HEPES, pH7.4 (TAAB Laboratories Equipment Ltd. Berks, U.K.) at 4°C for 2 hours, rinsed with 30mM HEPES at room temperature for 30 minutes and postfixed with 1%OsO_4/ 30mM HEPES, pH7.4 (TAAB Laboratories Equipment Ltd) at room temperature for 1 hour. Subsequently, dehydration was performed by soaking the samples in 50%, 70%, 80% ethanol at room temperature respectively for 15 minutes, followed by overnight incubation in 90% ethanol at 4°C and twice 15 minutes incubation in 100% ethanol. Conductive staining was performed using 10% phosphotungstic acid/ 100% ethanol. The samples were rinsed in 100% ethanol, mounted on aluminum stubs with carbon tapes, air-dried at room temperature for 10 minutes and subjected to scanning electron microscopic investigation using SU6600 low-vacuum electron microscope (Hitachi High-Tech, Tokyo, Japan) with accelerating voltage 7 kV, working distance 5 mm, vacuum condition 50 Pa using environmental secondary electron detector.

Assessment of the effect of minoxidil on hDP cells / iDPSCs

iDPSCs or cultured hDP cells (passage 0-1), originally microdissected from human scalp samples and cultured in Follicle Dermal Papilla Growth Medium (Promocell, Heidleberg, Germany), were cultured in DMEM: F12 (3:1) at 37^0C, 5% CO_2 with or without 10 μM minoxidil sulphate. For co-culture with hKCs, approximately 2.5 x 10^5 passage 3 hKCs

(CELLnTEC advanced cell systems, Bern, Switzerland) that had been expanded and cultured in CnT-57 (CELLnTEC) were seeded onto collagen coated permeable transwell inserts (Corning, Corning, NY) on iDPSC/hDP cell culture. The medium was changed on day 2 and on day 4 of these cultures, total RNA was extracted for real-time PCR as described above.

References for supplementary materials and methods

Hussein SM, Batada NN, Vuoristo S *et al*. (2011). Copy number variation and selection during reprogramming to pluripotency. *Nature* 471:58-62.

Imaizumi Y, Okada Y, Akamatsu W *et al*. (2012). Mitochondrial dysfunction associated with increased oxidative stress and alpha-synuclein accumulation in PARK2 iPSC-derived neurons and postmortem brain tissue. *Mol Brain* 5:35.

Mabuchi Y, Morikawa S, Harada S *et al*. (2013). LNGFR(+)THY-1(+)VCAM-1(hi+) Cells Reveal Functionally Distinct Subpopulations in Mesenchymal Stem Cells. *Stem Cell Reports* 1:152-65.

Nakao K, Morita R, Saji Y *et al*. (2007). The development of a bioengineered organ germ method. *Nat Methods* 4:227-30.

Okita K, Yamakawa T, Matsumura Y *et al*. (2012). An Efficient Non-viral Method to Generate Integration-Free Human iPS Cells from Cord Blood and Peripheral Blood Cells. *Stem Cells*:

Takahashi K, Tanabe K, Ohnuki M *et al*. (2007). Induction of pluripotent stem cells from adult human fibroblasts by defined factors. *Cell* 131:861-72.

Veraitch O, Kobayashi T, Imaizumi Y *et al*. (2013). Human induced pluripotent stem cell-derived ectodermal precursor cells contribute to hair follicle morphogenesis in vivo. *J Invest Dermatol* 133:1479-88.

Supplementary Table 1 Sequence of primers used for Quantitative PCR.

Gene (Gene accession no.)	Forward primer (5' to 3')	Reverse primer (5' to 3')
**GAPDH** (**NM_001256799)**	TGGAAATCCCATCACCATCTTC	CGCCCCACTTGATTTTGG
**PPARγ** **(NM_005037)**	GCTCTAGAATGACCATGGTTGAC	ATAAGGTGGAGATGCAGCTC
**LPL** **(NM_000237)**	GAGATTTCTCTGTATGGCACC	CTGCAAATGAGACACTTTCTC
**ADIPSIN** **(NM_001928)**	CAAGCAACAAAGTCCCGAGC	CCTGCGTTCAAGTCATCCTC
**OSTEOCALCIN** **(NM_199173)**	CCTCACACTCCTCGCCCTATT	CCCTCCTGCTTGGACACAAA
**BSP** **(NM_004967)**	AAACGAAGAAAGCGAAGCAGAA	GCTGCCGTTGCCGTTTT
**COL2** **(NM_001844)**	TCACGTACACTGCCCTGAAG	TGCAACGGATTGTGTTGTTT
**COL10** **(NM_000493)**	AATGCCCACAGGCATAAAAG	AGGACTTCCGTAGCCTGGTT
**AGGRECAN** **(NM_001135)**	GAAAGGCATCGTCTTCCATT	ACGTCCTCACACCAGGAAAC
**ALPL** **(NM_000478)**	ATTGACCACGGGCACCAT	CTCCACCGCCTCATGCA

**LEF1 (NM_001130713)**	CCCGATGACGGAAAGCAT	TCGAGTAGGAGGGTCCCTTGT
**WIF1 (NM_007191)**	TGGCATGGAAGACACTGCAA	GGCCTCAGGGCATGTATGA
**HEY1 (NM_012258)**	GCGCACGCCCTTGCT	GCCAGGCATTCCCGAAA
**WNT5A (NM_003392)**	TCCACCTTCCTCTTCACACTGA	CGTGGCCAGCATCACATC
**LRP4 (NM_002234)**	GGCACAGCCACTAGGTTTTAACA	GAAGGCCGAGGCAAGCA
**RGS2 (NM_002923)**	GACTGCAGACCCATGGACAA	AGAGATCCAGCGGGTGTTTG
**GUCY1A3 (NM_001130683)**	CCCAGTCCTCGCTGGTGAT	ACATGAAATGGAATGGAAATGTCTT
**BAMBI (NM_012342)**	CTCCCGTTTGCACTACAGCTT	CTTTGCAACCTGCCCCTTT
**BMP4 (NM_001202)**	GCCCGCAGCCTAGCAA	CGGTAAAGATCCCGCATGTAG
**IGF1 (NM_000618)**	AAGGAGGCTGGAGATGTATTGC	CGGACAGAGCGAGCTGACTT
**SOX2 (NM_003106)**	TGCGAGCGCTGCACAT	TTCTTCATGAGCGTCTTGGTTTT

**LAMC3 (NM_006059)**	GCTGGGCTGAGCGAGATG	CTGACAAGGTCTCGATGTCCTTCT
**TRPS1 NM_001282902**	TGAATCCCAGTCCCTGTTACG	GGCAATTGGCACAAAAAACAC
**MSX2 (NM_002449)**	GGGCCAAGGCGAAAAGA	GCAGCCATTTTCAGCTTTTCC
**KRT33A (NM_004138)**	TGCATGTGACAAGTCCACTG	ACCCAAATGTGTTGCAAGGC
**KRT75 (NM_004693)**	AGGACTGTGAGGCAGAACCTAGA	CCGTCGGAGCTCACTGGTA
**KRT82 (NM_033033)**	TCACCCCTGTCACCATCAATG	ATGCGAAACGGTTGTTGAGG
**KRT86 (NM_002284)**	AGCAAGTGTGAGGAGATGAAGG	TTGGAATTCTGGCACTTGGC

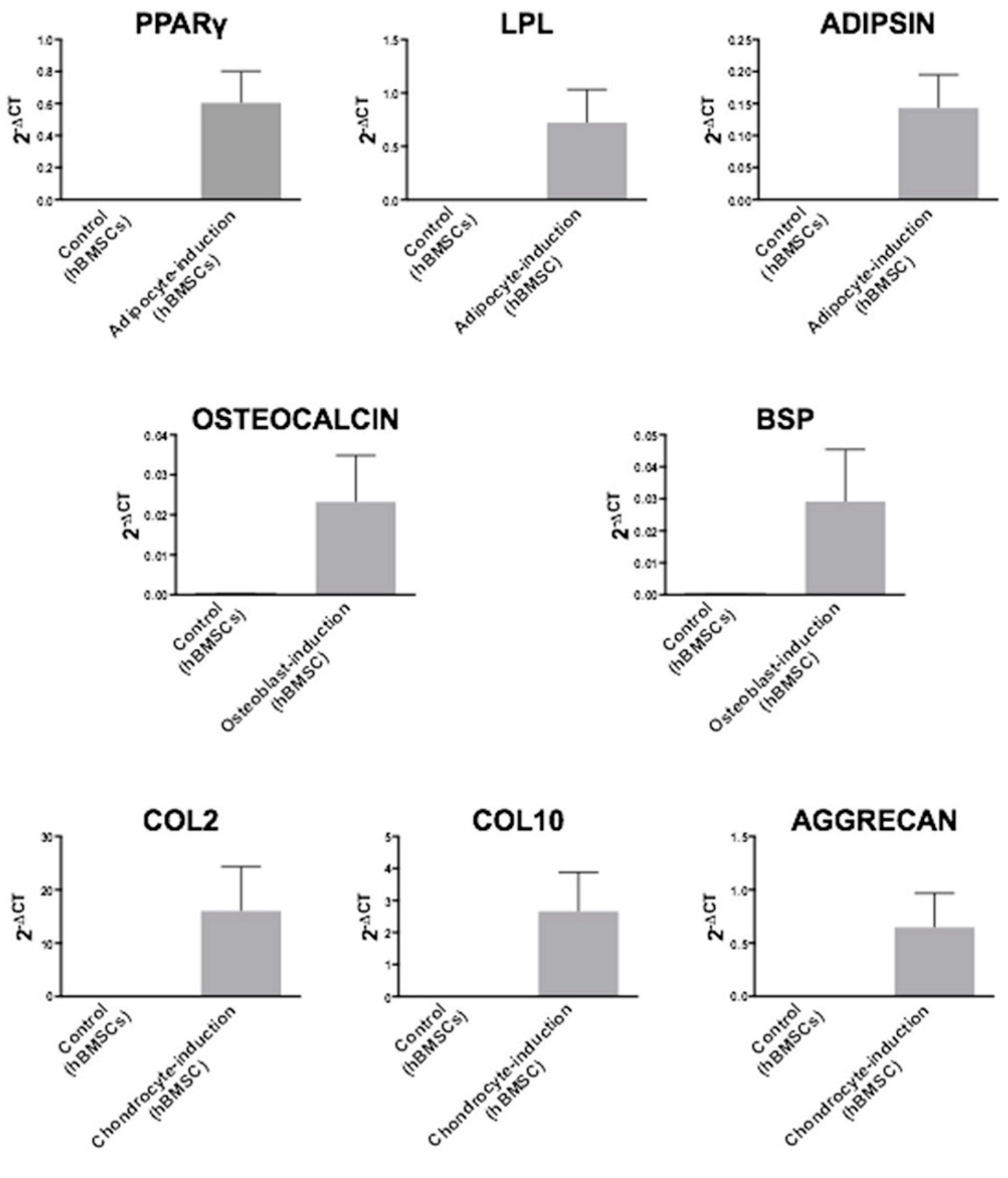

Supplementary Figure 1. Up-regulation of three mesenchymal lineage genes in the hBMSC-derived LNGFR(+)THY-1(+) subset

Fold changes in gene expression levels of osteoblast, adipocyte, and chondrocyte lineage genes were greater in the hBMSC-derived LNGFR(+)THY-1(+) cell population.

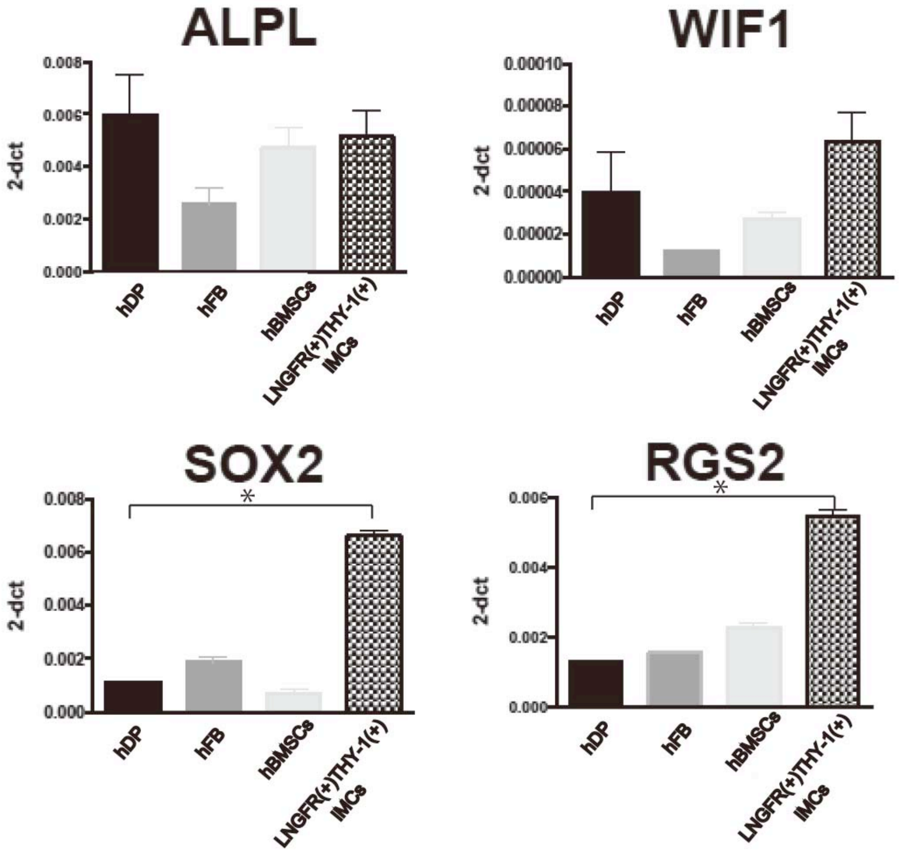

Supplementary Figure 2. LNGFR(+)THY-1(+) iMCs innately express higher levels of key DP signature genes compared to other mesenchymal cells

Compared to human dermal papilla cells (hDP), human fibroblasts (hFBs), human bone marrow stromal cells (hBMSCs), and the LNGFR(+)THY-1(+) iMC subset expressed higher levels of DP signature genes known to be correlated with intrinsic properties, suggesting that use of LNGFR(+)THY-1(+) iMCs would be advantageous for iDPSC induction (*$P < 0.05$).

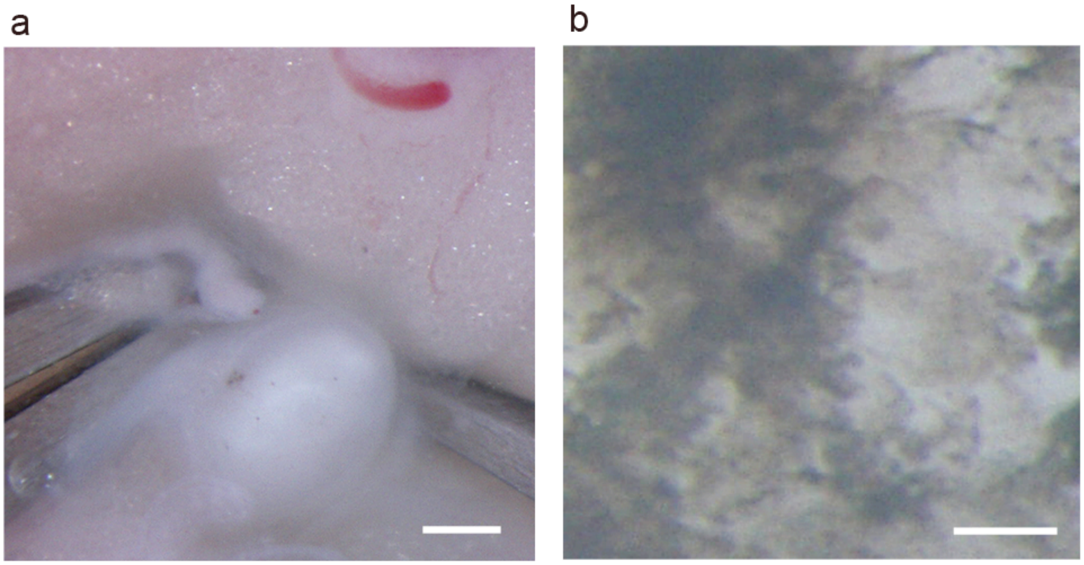

Supplementary Figure 3. The result of patch assay using cultured normal adult hKC and hDP cells

(a) Unlike a cystic structure formed by mouse cells, only a barely detectable tiny fibrotic structure was observed. Size comparison with extra fine watchmaker's forceps tells the size of the regenerated structure. **(b)** No hair-like structure was noted after microdissection. Scale bar = 200 μm.

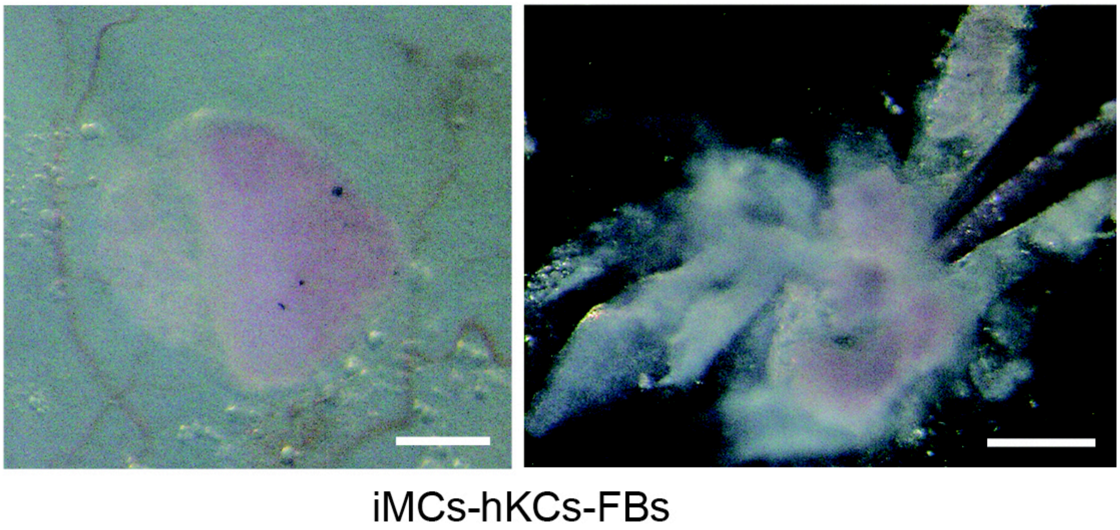

Supplementary Figure 4. Co-transplantation of human keratinocytes and non-RA-DPAC-treated iMCs subcutaneously into immunedeficient mice did not form hair follicle-like structures

When co-grafted with human keratinocytes, non-RA-DPAC-treated iMCs formed retention cysts (left panel). However, hair follicle structures were not observed following microdissection (right panel). Scale bar = 200 µm.

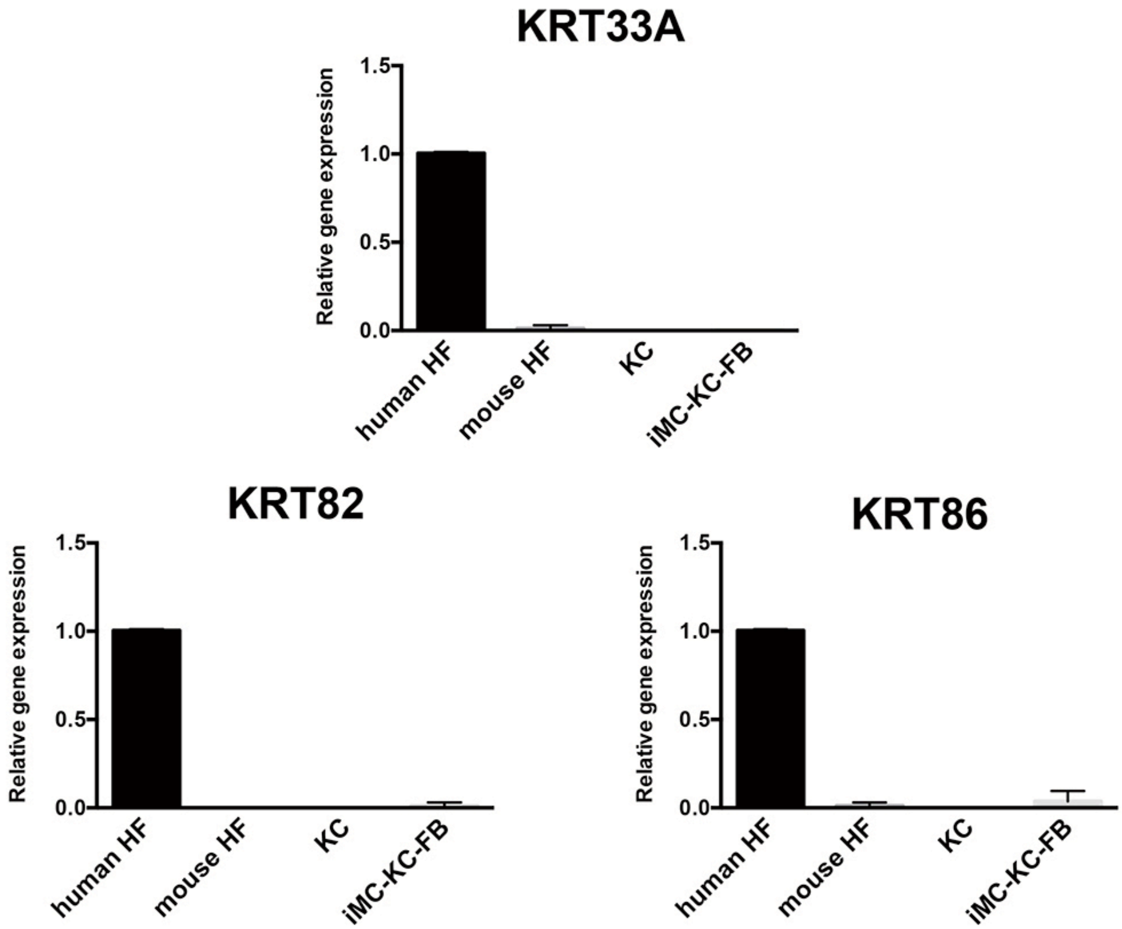

Supplementary Figure 5. Human hair specific keratin gene expression was not detected in the area where human keratinocytes, iMCs and fibroblasts were co-transplanted.

Unlike in the area containing hair-like structure induced by co-transplantation of KC-DP-FB or KC-iDPSC-FB (presented in Figure 4f), up-regulation of hair specific keratin was not detected in the area where human keratinocytes, iMCs and fibroblasts were co-transplanted. “KC” in X-axis indicates the value obtained from cultured human adult interfollicular epidermal keratinocytes (second negative control)
